# Limited Role of Malonic Acid in Sulfuric Acid–Dimethylamine New Particle Formation

**DOI:** 10.1021/acsomega.3c01643

**Published:** 2023-05-19

**Authors:** Sandra K.W. Fomete, Jakub Kubečka, Jonas Elm, Coty N. Jen

**Affiliations:** † Department of Chemical Engineering, Carnegie Mellon University, Pittsburgh, Pennsylvania 15213, United States; ‡ Center for Atmospheric Particle Studies, Carnegie Mellon University, Pittsburgh, Pennsylvania 15213, United States; § Department of Chemistry, Aarhus University, Langelandsgade 140, 8000 Aarhus C, Denmark

## Abstract

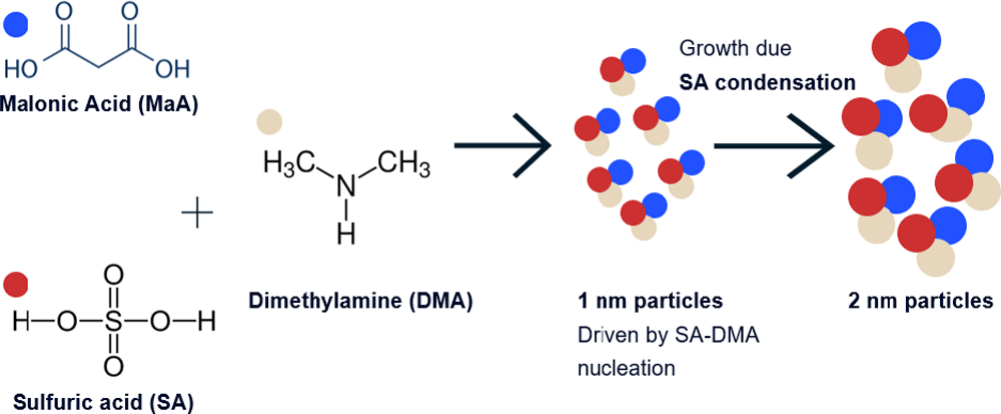

Aerosols play an important role in climate and air quality; however, the mechanisms behind aerosol particle formation in the atmosphere are poorly understood. Studies have identified sulfuric acid, water, oxidized organics, and ammonia/amines as key precursors for forming aerosol particles in the atmosphere. Theoretical and experimental investigations have indicated that other species, such as organic acids, may be involved in atmospheric nucleation and growth of freshly formed aerosol particles. Organic acids, such as dicarboxylic acids, which are abundant in the atmosphere, have been measured in ultrafine aerosol particles. These observations suggest that organic acids may contribute to new particle formation in the atmosphere but their role remains ambiguous. This study examines how malonic acid interacts with sulfuric acid and dimethylamine to form new particles at warm boundary layer conditions using experimental observations from a laminar flow reactor and quantum chemical calculations coupled with cluster dynamics simulations. Observations reveal that malonic acid does not contribute to the initial steps (formation of <1 nm diameter particle) of nucleation with sulfuric acid-dimethylamine. In addition, malonic acid was found to not participate in the subsequent growth of the freshly nucleated 1 nm particles from sulfuric acid-dimethylamine reactions to diameters of 2 nm.

## Introduction

Atmospheric new particle formation (NPF), which comprises nucleation and subsequent growth of particles, contributes significantly to the formation of cloud condensation nuclei (CCN), thus affecting the Earth’s radiative budget.^1,2^ Studies have shown that nucleation, the process where trace amounts of precursor vapors react or cluster to form stable particles, produces approximately 50% of the global CCN.^3−7^ Understanding nucleation and initial particle growth is crucial to better predict the impact of aerosol particles on clouds and the climate. Sulfuric acid nucleation with ammonia and amines in the atmosphere has been widely established by several studies as important pathways for nucleation in the polluted boundary layer.^8−13^ However, the sulfuric acid concentrations in the atmosphere are generally too low to enable the growth of clusters to CCN sizes, leaving atmospherically abundant organic acids as plausible agents for the growth of nucleated clusters.

Organic dicarboxylic acids are highly oxidized molecules that have been measured in ultrafine aerosol particles (diameters < 100 nm) around the world.^14−22^ As a result, these molecules may contribute to the observed nucleation and growth rates in the atmosphere. Dicarboxylic acids in the atmosphere come from various biogenic and anthropogenic sources.^23,24^ The primary sources of dicarboxylic acid vapors in the atmosphere include animal waste, chemical plants, wildfires, tobacco smoke, and others.^25−28^ Atmospheric dicarboxylic acids are also generated via secondary pathways such as photo-oxidation of volatile organic precursors.^29,30^ Though dicarboxylic acids have been measured in ultrafine particles, their role in NPF from gas-phase reactions in the atmosphere is still not well known.

Computational chemistry studies predict that dicarboxylic acids can offer some stabilization to sulfuric acid clusters.^31−34^ Dicarboxylic acids are very polar and contain sites for hydrogen bonding with nucleation precursors such as sulfuric acid and amines. In addition, small dicarboxylic acids are intermediate volatility compounds, which make them potential candidates for NPF in the atmosphere.^35,36^ On a molecular level, the mechanisms through which small dicarboxylic acids, such as malonic acid, participate in the initial steps of nucleation and growth of clusters from sulfuric acid-amine reactions have not been investigated extensively. Sulfuric acid and ammonia/amine reactions are responsible for nucleation events observed in various parts of the world.^8,11−13,37−48^ Computational studies of cluster formation involving sulfuric acid, ammonia, and malonic acid have previously been performed by Zhang et al.^34^ Extremely cold temperatures (218 K) were required for malonic acid to enhance NPF in any appreciable amount. The computational study of sulfuric acid, dimethylamine, and malonic acid clusters by Wang et al. found that malonic acid could potentially participate in sulfuric acid-dimethylamine nucleation.^33^ The nucleation rate of the sulfuric acid-dimethylamine-malonic acid system was found to be in between that of the sulfuric acid-dimethylamine-water and sulfuric acid-ammonia-water systems at 278 K. However, only 40 low-energy structures based on the PM7 semi-empirical level of theory were considered, which makes it possible that the global minimum structure was not observed. In addition, Wang et al. applied the RI-MP2/cc-pVTZ level of theory to calculate the binding energies of their clusters. It is well known that MP2 overestimates the binding energies of atmospheric molecular clusters^49^ and thereby their calculated cluster stabilities are expected to be overestimated.

Experimental evidence of the role of small dicarboxylic acids is still lacking. Specifically, malonic acid is abundant in the atmosphere and has a low vapor pressure (1.2 × 10^–3^ Pa at 298 K).^50^ Previous measurements from rural Lamont, OK show malonic acid at 10^7^–10^9^ cm^–3^, higher than sulfuric acid concentrations of 10^5^–10^7^ cm^–3^.^51^ Fang et al. measured 10^7^–10^9^ cm^–3^ of malonic acid at Pingyuan rural site and observed a correlation of nucleation rates with concentrations of diacids.^52^ The authors also observed clusters of small diacids, including malonic acid, and concluded that malonic acid has the potential to enhance the initial steps of sulfuric acid-dimethylamine particle formation and growth.

This study presents experiments performed in a laminar flow reactor to investigate the role of malonic acid in the initial steps of nucleation and growth of freshly formed clusters in the presence and absence of sulfuric acid and stabilizing bases such as dimethylamine. A chemical ionization mass spectrometer (CIMS) and a versatile water condensation particle counter (vWCPC) were used to examine molecular clusters and particle number concentrations, respectively. Complimentary quantum chemical calculations were also conducted on clusters consisting of sulfuric acid, dimethylamine, and malonic acid. Based on the calculated thermochemistry, the cluster population dynamics simulations are performed and compared to the experimental results.

## Experimental Section

**Figure 1:**
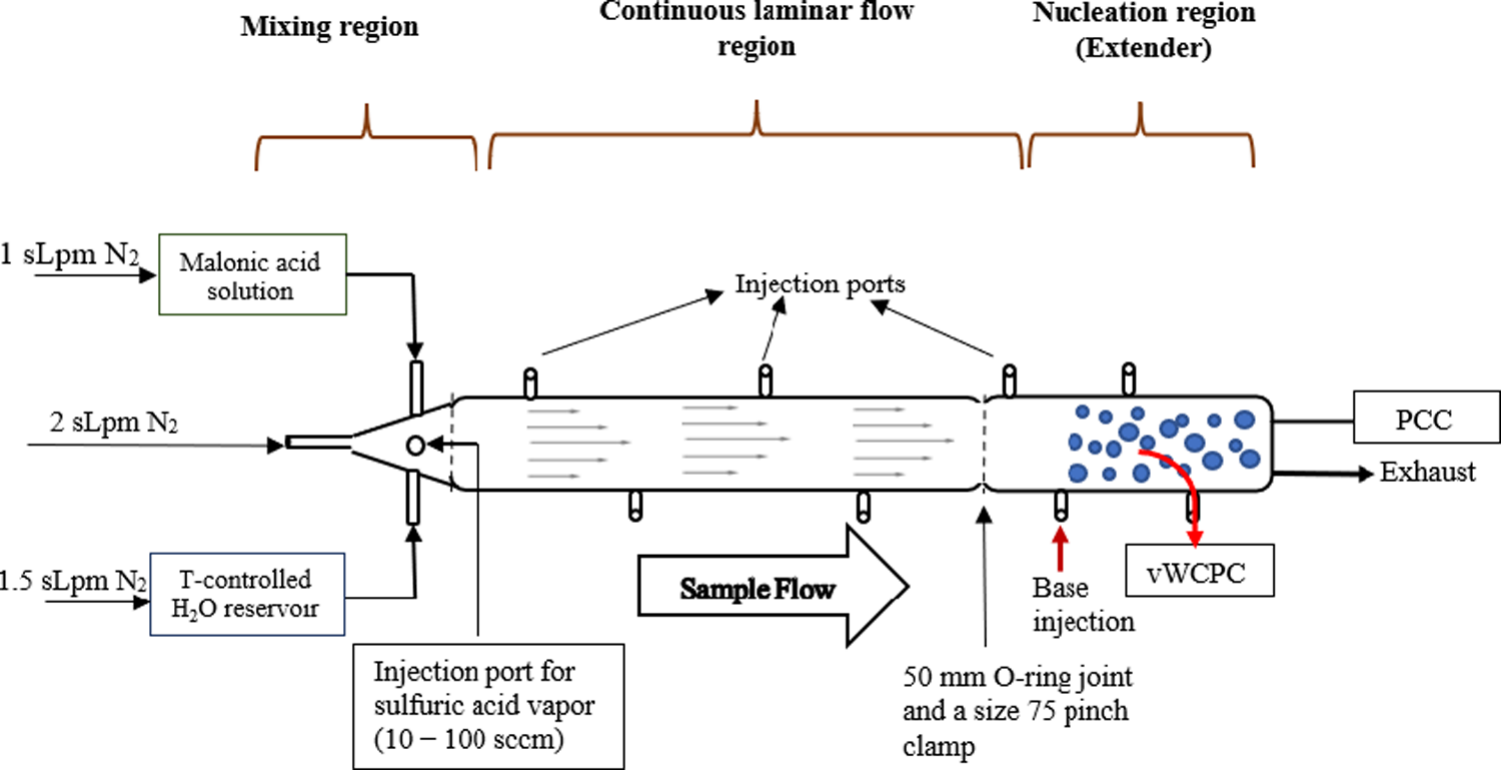
Schematic of the malonic acid flow tube reactor. The reactor is set up in a vertical position during experiments.

Malonic acid (MaA) and dimethylamine (DMA) were reacted with gaseous sulfuric acid to produce freshly formed clusters in a clean, laminar flow reactor. This glass flow reactor has been previously described in Fomete et al., with key details repeated here.^53^ Malonic acid vapor, humified nitrogen, and nitrogen carrier gas are injected into the top of the reactor (Figure 1). 2.0 sLpm of nitrogen carrier gas is supplied to the reactor from an ultra-high purity liquid nitrogen tank (99.999% purity, Matheson). Malonic acid at 1.0 sLpm injected into the reactor is produced by flowing nitrogen gas over a reservoir containing 15 wt % malonic acid in HPLC-grade water. 1.5 sLpm of humidified nitrogen is also injected into the reactor by flowing nitrogen over a reservoir containing HPLC-grade water. The flow reactor was operated at 1 atm, 20% RH, 300–303 K, and a total flow rate of 4.5–4.6 sLpm.

Sulfuric acid was injected into the mixing region of the reactor, shown in Figure 1. Sulfuric acid concentrations in this study were maintained at a factor of ∼10 lower than malonic acid to mimic what has previously been observed in the field.^51,52^ Dimethylamine (DMA) was injected into the reactor’s nucleation region, resulting in a nucleation reaction time of ∼10 s. This time was determined using the center line flow velocity (7.3 m/s) calculated via computational fluid dynamics.^54^ Gas-phase DMA was produced by passing nitrogen over a temperature-controlled (300 K) permeation tube containing DMA (40 wt % solution in water, Fischer Scientific). The gas-phase concentration of DMA was varied (0–33 ptv) by the use of a double-stage dilution system.^53^ The range of DMA concentrations studied here also follows those previously observed in the atmosphere.^55−57^ DMA was injected into the centerline of the flow in the reactor to minimize vapor wall losses.^53^

The flow reactor was continuously purged with nitrogen, water, and malonic acid vapors when experiments were not being conducted to minimize contamination that may interfere with the targeted nucleation reactions.^53^ Baseline conditions were conducted each day to ensure clean and repeatable reaction conditions in the flow reactor before an experiment.^9^ These baseline measurements, shown in Figure S1a,b (SI-1) in the supplementary information (SI), involve measuring the malonic acid dimer concentrations ([MaA_2_]) at a given malonic acid monomer concentration ([MaA_1_] at 103 amu) and the sulfuric acid dimer concentrations ([SA_2_] at 195 amu) at a given sulfuric acid monomer concentration ([SA_1_] at 97 amu) without the addition of DMA. In addition, baseline 1-nm particle concentrations at various [MaA_1_] were taken and are shown in Figure S2 in SI-2. SA_*i*_ and MaA_*i*_ represent clusters containing *i* sulfuric acid or malonic acid molecules and were detected without any other base or water ligands. The heterodimer of malonic acid and sulfuric acid, which was detected without any attached ligands, is represented as MaA_1_·SA_1_. Other ligand molecules were likely attached to these clusters but evaporated upon ionization and/or measurement with the mass spectrometer.^44^

A previously described custom-built transverse chemical ionization inlet, coupled to an atmospheric pressure, long time-of-flight mass spectrometer (Tofwerk AG), called the Pittsburgh Cluster CIMS (PCC), was attached in-line with the malonic acid flow reactor to measure concentrations of gases and freshly formed clusters.^58^ Malonic and sulfuric acid concentrations, as well as the neutral clusters formed in the flow reactor, were measured in negative ion mode with the PCC using acetate as the chemical ionization reagent ion. Acetate ion, compared to nitrate, was chosen to ensure efficient ionization of malonic acid. The dominant acetate reagent ions are H_2_O · CH_3_CO_2_^–^ (∼6 × 10^3^ Hz), CH_3_CO_2_H · CH_3_CO_2_^–^ (∼1 × 10^5^ Hz), and CH_3_CO_2_^–^ (∼6 × 10^4^ Hz). The concentration of DMA in the sample flow was measured in positive ion mode using hydronium ions, (H_2_O)_1 – 2_ · H_3_O^+^. See SI-3 for an explanation on how the acid and amine concentrations were calculated. The important flows and voltage parameters used in the custom-built inlet of the PCC were chosen in order to result in a 25 ms chemical ionization reaction time.^53^ The systematic uncertainty of the PCC is estimated to be a factor of 2.^58^ In addition, a versatile water condensation particle counter (vWCPC, TSI 3789) was connected through one of the side ports in the nucleation region of the reactor to detect the total particle number concentration of freshly nucleated particles.^59^ The vWCPC operated at 1.5 sLpm total flow to minimize diffusional losses within the sampling line. The operating temperatures of the vWCPC were set to 1.5, 90, and 22 °C for the conditioner, initiator, and moderator stages, respectively, to measure particles down to ∼1 nm (50% cut-off size, *d*_50_). For the *d*_50_ of 2 nm, the temperatures were 10, 90, and 22 °C for the conditioner, initiator, and moderator stages respectively.

## Computational Details

A subset of the relevant SA_0–3_DMA_0–3_MaA_0–1_ clusters was chosen as a test case to simulate cluster formation of the SA–DMA–MaA system (see SI-4 for the list of clusters). Atmospheric Cluster Dynamics Code (ACDC),^60^ representing the cluster birth-death equations, was used to determine concentrations of various formed clusters. ACDC incorporates all possible collision and evaporation patterns for all monomers and clusters. At each given initial monomer concentration, a 10 s simulation was performed at standard condition (298.15 K and 1 atm) to mimic the flow tube experiment. Wall losses for flow tube experiments with a tube radius of 2.5 cm were also included (see ACDC for more details). The clusters that were allowed to grow out of the simulation system were set to be SA_*i*≥3_DMA_*j*≥3_MaA_*k*≥0_. These clusters are assumed to be stable enough to grow further into particles. The formation rate of outgrowing clusters thus defines the particle formation rate. Clusters that are not within our simulation scheme and within the outgrowing clusters are assumed to be unstable and fragment back into the simulation system. The simulation scheme is quite small. However, the calculated particle formation rate will have only a small offset but the studied trends should be correct. In other words, the simulated system is large enough to support the experimental data and reveal the stability of clusters containing malonic acid, as well as its effect on the atmospheric NPF of SA–DMA system.

The collision probabilities of each cluster type are calculated from kinetic gas theory.^60^ The evaporation probabilities are calculated from the balance equation and cluster binding free energies obtained from quantum chemical calculations.^60^ The structures and energies of the SA–DMA clusters were taken from Kubečka et al.^61^ The remaining clusters containing one MaA were constructed through the same configurational sampling protocol, and the binding free energies were evaluated at the same level of theory (i.e., DLPNO–CCSD(T)^62−66^/aug-cc-pVTZ//ωB97X-D^67^/6-31++G(d,p)) and using the same computational software (i.e., ABCluster,^68,69^ XTB,^70,71^ Gaussian,^72^ and ORCA.^73,74^ The DLPNO–CCSD(T)/aug-cc-pVTZ//ωB97X-D/6-31++G(d,p) level of theory has been thoroughly benchmarked both with regards to the structures^75,76^ and binding energies.^49,77^ This methodology is generally recommended for atmospheric cluster formation studies by our group^78^ and others.^79^ More technical details are given in Kubečka et al.^61^ The identified structure coordinates and binding free energies of all clusters are presented in SI-4.

## Results and Discussion

**Figure 2:**
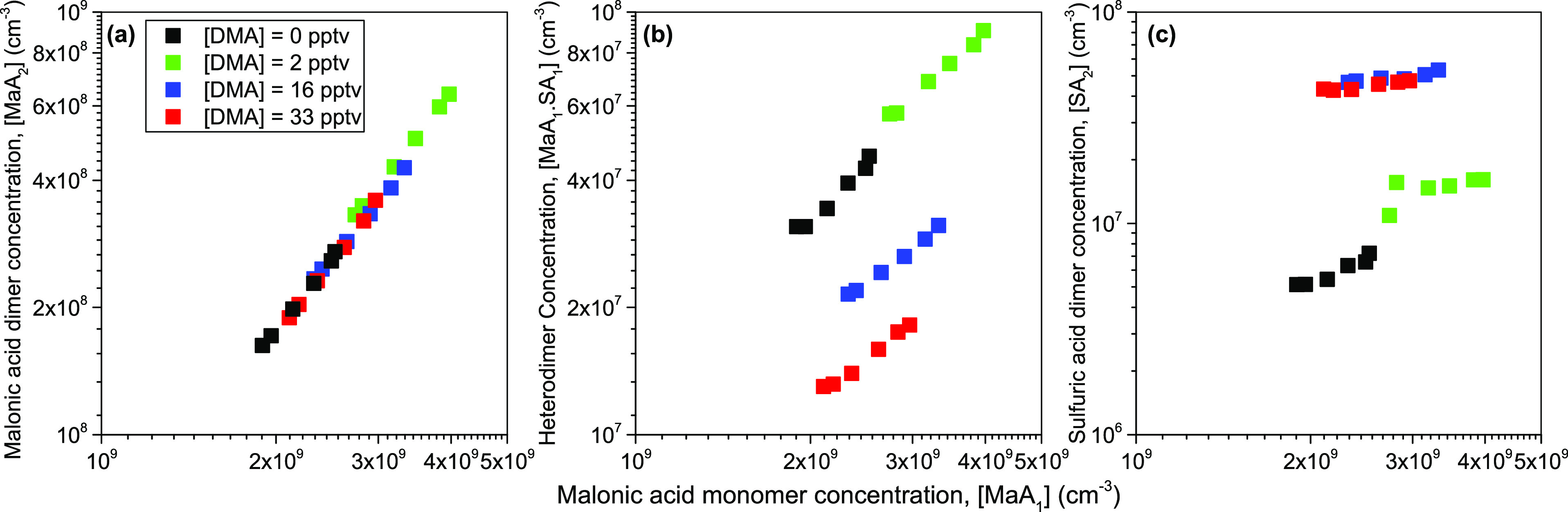
(a) Measured malonic acid dimer concentration ([MaA_2_]) vs malonic acid monomer concentration ([MaA_1_]), (b) malonic acid and sulfuric acid heterodimer ([MaA_1_·SA_1_]) vs [MaA_1_], and (c) sulfuric acid dimer concentration ([SA_2_]) vs [MaA_1_]. Each color represents a different [DMA_1_] between 0 and 33 pptv. The sulfuric acid concentration ([SA_1_]) was constant at 3 × 10^8^ cm^–3^.

Figure 2 presents the measured dimer concentrations of malonic acid ([MaA_2_]), the heterodimer of malonic acid and sulfuric acid ([MaA_1_·SA_1_]), and the sulfuric acid dimer concentration ([SA_2_]) as a function of malonic acid concentration ([MaA_1_]). Each color represents a different [DMA_1_] ranging from 0 to 33 pptv, and [SA_1_] was held constant at 3 × 10^8^ cm^–3^. For Figure 2a, most malonic acid dimers were detected at 207 *m*/*z* (C_3_H_4_O_4_·C_3_H_3_O_4_^–^), with small amounts (<5%) detected with acetate ligands at 267 and 327 *m*/*z*. It can be seen in Figure 2a that increasing the [DMA_1_] from 0 to 33 pptv did not affect the measured [MaA_2_]. [MaA_2_] displays a squared dependency on [MaA_1_], suggesting that its formation depends primarily on the collision of two MaA_1_ with no involvement of DMA. This implies that amines, such as DMA do not enhance the formation of MaA_2_ and ternary nucleation of MaA-DMA-H_2_O in the absence of sulfuric acid is very unlikely at 300 K. This is corroborated by quantum chemical calculations as the binding free energy of malonic acid interacting with DMA (−4.0 kcal/mol) is relatively high compared to SA_1_·DMA_1_ (−11.4 kcal/mol). This implies that the [MaA_1_·DMA_1_] cluster will have a very low gas-phase concentration.

Figure 2b shows the concentration of the heterodimer of malonic acid and sulfuric acid [MaA_1_·SA_1_] vs [MaA_1_] for [DMA_1_] = 0–33 pptv and [SA_1_] = 3 × 10^8^ cm^–3^. The [MaA_1_·SA_1_] increases with increasing [MaA_1_] when [DMA_1_] is kept constant. In addition, [DMA_1_] from 0 to 2 pptv, the [MaA_1_·SA_1_] increases due to increased [MaA_1_] as evidenced by a continuation of the [DMA_1_] = 0 pptv curve. The computed free energies and the results of Wang et al.^33^ and our theoretical calculations show that hydrogen bonding is possible between sulfuric and malonic acid in this acid heterodimer which explains why MaA_1_·SA_1_ was experimentally measured (see Figure S3 in SI-4 for the heterodimer structure). As [MaA_1_] is increased in the reactor, more malonic acid is available to collide and cluster with sulfuric acid to form MaA_1_·SA_1_. However, when [DMA_1_] is increased in the reactor, the formation of MaA_1_·SA_1_ is reduced. For example, at [MaA_1_] = 3 × 10^9^ cm^–3^, [MaA_1_·SA_1_] decreases from 6.2 × 10^7^ to 1.8 × 10^7^ cm^–3^ when [DMA_1_] is increased from 2 and 33 pptv. This suggests that the presence of DMA hinders the formation of MaA_1_·SA_1_ in the reactor. Although the theoretical simulations resulted in lower [MaA_1_·SA_1_] (see Figure S4a), the simulations reproduce similar DMA hindering effects. This hindrance can be understood from the free energies of these formed clusters. The reaction free energy for forming MaA_1_·SA_1_ is −5.6 kcal/mol, whereas the formation of SA_1_·DMA_1_ is −11.4 kcal/mol. This implies that the [SA_1_·DMA_1_] will build up over time, with DMA scavenging the free sulfuric acid available to form MaA_1_·SA_1_.^9,80^ Cluster growth of MaA_1_·SA_1_ to larger sizes with and without DMA is not favorable as no larger malonic acid clusters (MaA_*i*≥2_) containing either sulfuric acid and/or DMA were measured.

Figure 2c shows [SA_2_] vs [MaA_1_], where [DMA_1_] = 0–33 pptv and [SA_1_] = 3 × 10^8^ cm^–3^. As previously shown, DMA enhances sulfuric acid dimer formation, which is seen by increased sulfuric acid dimer concentrations with increasing [DMA_1_].^8,9,55,80−83^ Notice that the concentration of sulfuric acid dimer is slightly lower at [DMA_1_] = 33 pptv than at 16 pptv. This is likely due to the loss of more sulfuric acid dimers to sulfuric acid dimer–dimer coagulation when [DMA_1_] is very high.^9^ Generally, increasing malonic acid monomer concentrations in Figure 2c leads to a slight increase in [SA_2_]. Due to the narrow range of [MaA_1_] explored in Figure 2, more measurements are needed at higher and lower [MaA_1_] to better examine its effect on the aforementioned cluster concentrations. Figure S4b shows that the simulated [SA_2_] does not change with [MaA_1_]. In addition, [SA_2_] decreases with [DMA_1_] because SA_1_DMA_1_ clusters are more stable than SA_2_ and DMA also hinders the SA_2_ formation. Figure S4c shows the concentration of all simulated clusters containing two SA, i.e., [N_2_] = [SA_2_DMA_0–3_MaA_0–1_] at different [DMA_1_]. Simulated [N_2_] reproduces well the trends observed in Figure 2c which shows the [SA_2_]. Note, measured [SA_2_] includes various ligands (e.g., SA_2_·(H_2_O)_*x*_, SA_2_·MaA_1_, and SA_2_·(DMA)_*x*_) which then fragment within the PCC due to the ionization; leaving the pure SA cluster ion to be detected.

Compared to our experimental results, Wang et al.^33^ reported the formation of a stable MaA_1_·SA_1_·DMA_1_ cluster from computational chemistry calculations, which predicts that malonic acid can form hydrogen bonds with sulfuric acid and a stable cyclic ring structure when DMA is added. However, MaA_1_·SA_1_·DMA_1_ was not observed experimentally, maybe due to the short lifetime of its ion form. Also, large MaA–SA clusters or MaA–DMA clusters were not measured. Therefore, it is not likely that MaA_1_·SA_1_·DMA_1_ contributes to the initial stages of nucleation as it, and even the large clusters containing more MaA, SA, and/or DMA, were not measured.

Based on our quantum chemical calculations and in agreement with the reported evaporation rates by Wang et al., the SA_1_·DMA_1_ cluster is more stable (Δ*G* = −11.4 kcal/mol) than MaA_1_·DMA_1_ cluster (Δ*G* = −4.0 kcal/mol).^33^ In MaA_1_·DMA_1_, no proton transfer occurs between the malonic acid and DMA, and thereby the cluster is held together purely by hydrogen-bonded interactions, as opposed to electrostatic in the SA_1_·DMA_1_ cluster. This explains the shorter lifetime of MaA_1_·DMA_1_ compared to SA_1_·DMA_1_.^33,84^ For the experiments carried out in this study, neither MaA_1_·DMA_1_ nor SA_1_·DMA_1_ were measured by the PCC due to evaporation of the base upon ionization.^33,84^ However, larger clusters containing only sulfuric acid and DMA were measured. This suggests that SA_1_·DMA_1_ grows rapidly to larger clusters faster than it evaporates. In contrast, clusters do not grow via the addition of MaA_1_·DMA_1_, as this cluster quickly evaporates. Although it is also possible that MaA_1_ could be lost from larger clusters upon ionization, overall, the initial stages of cluster stabilization and growth appear to be driven primarily by SA–DMA interactions.

**Figure 3:**
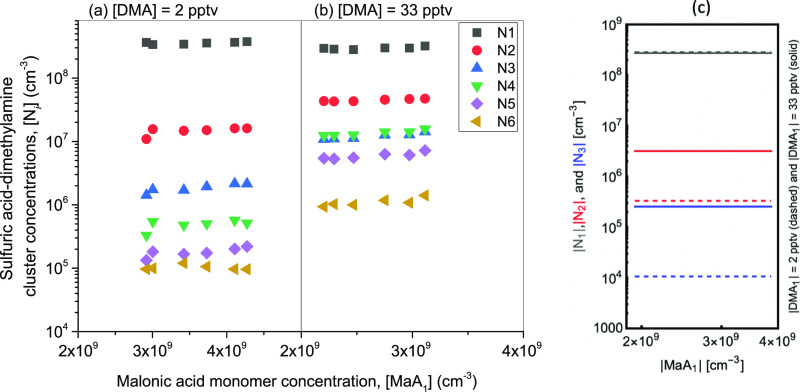
Measured concentrations of clusters with *i* sulfuric acid molecules ([N_*i*_]) at varying malonic acid concentration ([MaA_1_]) where (a) is low [DMA_1_] = 2 ppt and (b) high [DMA_1_] = 33 ppt. [SA_1_] held constant at 3 × 10^8^ cm^–3^. (c) Simulated SA–DMA cluster concentrations ([N_*i*_]) at varying malonic acid concentration ([MaA_1_]) and DMA concentration.

Figure 3 shows the measured and simulated concentrations of SA_*i*_DMA_0–*i*_MaA_0–1_ (N_*i*_) clusters as a function of [MaA_1_] at various [DMA_1_]. [SA_1_] was held constant at 3 × 10^8^ cm^–3^ with up to 5% increase throughout the experiment due to the passivation of the sulfuric acid on the injection setup. Note, N_*i*_ represents clusters containing *i* sulfuric acid molecules with equal or fewer base molecules (clusters are typically unstable with more base molecules and one base molecule likely evaporates upon ionization),^83,84^ up to one MaA (assuming that more MaA destabilizes the clusters), and any number of water molecules. For a given [DMA_1_], an increase in [MaA_1_] does not lead to a noticeable increase in various N_*i*_ clusters, as shown in Figure 3a,b. This trend is confirmed by the cluster dynamics simulations shown in Figure 3c. Note, the slight increase in [N_*i*_] observed in Figure 3 is likely due to 5% variation in [SA_1_]. However, increasing [DMA_1_] leads to a significant increase in [N_*i*_] (see Figure 3a,b). A relatively similar trend is again reproduced in the dynamics simulation (Figure 3c), and it has been previously observed too.^83^ This is due to the fact that sulfuric acid is in excess compared to DMA during the measurements, and DMA stabilizes sulfuric acid dimers with barrierless growth to ∼1 nm particle sizes.^9,80,85^ Note, when [DMA_1_] = 33 pptv, the measured sulfuric acid tetramer concentration is greater than [N_3_] at high [DMA_1_] due to the formation of N_4_ from N_2_. At high [DMA_1_], the concentration of available N_2_ is high, which would result in a similar number of N_2_–N_1_ and N_2_–N_2_ collisions to form comparable [N_3_] and [N_4_]. Regardless, the observations of Figure 3 strongly suggest that the malonic acid likely does not contribute to the initial steps of SA–DMA atmospheric NPF.

The key uncertainty in the PCC measurements is the ionizability of large clusters containing sulfuric acid, malonic acid, and DMA by acetate. For SA–DMA, acetate has been shown to detect more types of large clusters compared to nitrate reagent ion.^83^ However, to our knowledge, no previous studies have measured SA–MaA–DMA clusters using acetate ions. Malonic acid is still a much stronger acid than acetic acid and should therefore be ionized by acetate ions. Therefore, since no malonic acid clusters with any number of DMA and/or sulfuric acids, except for the heterodimer, were measured, this implies that these clusters were not formed in the reactor.

**Figure 4:**
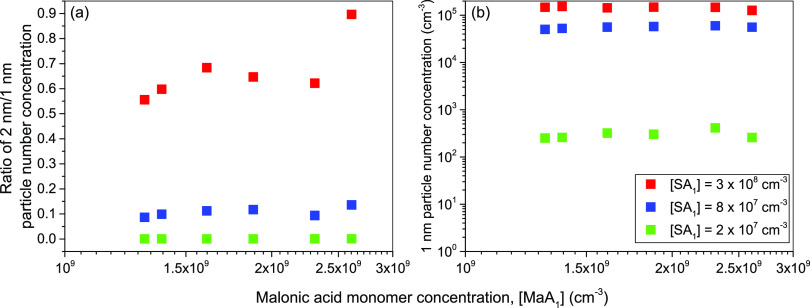
(a) Ratio of >2 to >1 nm particle number concentrations as a function of the malonic acid concentration ([MaA_1_]). (b) Number concentration of >1 nm particles vs [MaA_1_] for sulfuric acid concentrations of 2 × 10^7^ cm^–3^ (green), 8 × 10^7^ cm^–3^ (blue), and 3 × 10^8^ cm^–3^ (red). [DMA_1_] was kept constant at 7 pptv.

To explore if malonic acid instead plays a role in particle growth, experiments were carried out in which the concentrations of particles >1 nm were measured by the vWCPC. Figure 4a shows the ratio of measured 2 to 1 nm particle number concentrations as a function of [MaA_1_] for different sulfuric acid concentrations injected into the reactor with [DMA_1_] kept constant at 7 pptv. For a given sulfuric acid concentration, the fraction of >2 to >1 nm particle number concentration remains constant with varying [MaA_1_]. However, increasing the concentration of sulfuric acid from 2 × 10^7^ to 3 × 10^8^ cm^–3^ leads to an increase in the fraction of >2 to >1 nm particle number concentrations from ∼0 to 65%. The observed increase in the fraction of >2 nm particles with increasing [SA_1_] is due to the condensation of sulfuric acid. In Figure 4b, the particle number concentration for >1 nm at a given sulfuric acid concentration does not change with an increase in malonic acid concentration. This suggests that the malonic acid does not help form 1 nm particles or grow them to larger sizes. The ACDC simulations also show that the particle formation rate is independent of the malonic acid concentrations over a wide range of concentrations (see Figure S5), which further corroborates the experimental findings. Error bars are not shown in Figure 4a because only one set of measurements for 2 nm particles at [DMA_1_] = 7 pptv was obtained. Uncertainty in the absolute particle number concentrations due to uncertainty in activation efficiency will not affect the conclusions of the vWCPC results as the trends of particle number concentrations are consistent for different sulfuric acid and DMA concentrations. Overall, these results suggest two conclusions for systems where malonic acid concentrations are much greater than sulfuric acid concentrations: (1) malonic acid does not help nucleate 1 nm particles or grow 1 nm particles to 2 nm, and (2) for sub-2 nm particle sizes, nucleation and subsequent particle growth is driven mainly by sulfuric acid.

## Conclusions

The effect of malonic acid on sulfuric acid-dimethylamine nucleation and initial particle growth has been examined experimentally and using modeling supported by quantum chemical calculations. Malonic acid is a highly oxidized, acidic organic molecule that was previously predicted to enhance sulfuric acid new particle formation rates. However, this study shows that malonic acid does not enhance the formation of stable sulfuric acid-dimethylamine clusters or the growth of clusters from 1 to 2 nm. For sub-2 nm particles, sulfuric acid condensation is found to be the dominant pathway for nucleation and initial particle growth even in the presence of high malonic acid concentrations compared to sulfuric acid concentrations. Small organic acids, such as malonic acid likely contribute to the growth of larger particles >2 nm, as these organic acids have been measured in larger ultrafine particles globally. Further experiments should be conducted to examine the role of malonic acid on the growth of particles >2 nm. The results from this study are useful in narrowing the range of potential compounds that enhance sulfuric acid new particle formation in the atmosphere.

## Supporting Information Available

Additional details for daily baseline measurements and methods for calculating cluster concentrations (PDF)
