# Supplementary material for: Limited Role of Malonic Acid in Sulfuric Acid–Dimethylamine New Particle Formation
Source: ACS Omega. 2023 May 19;8(22):19807–15. doi: 10.1021/acsomega.3c01643 (PMC10249388; doi:10.1021/acsomega.3c01643)
Supplement: Supplementary file 1 — ao3c01643_si_001.pdf [file ao3c01643_si_001.pdf]

Limited Role of Malonic Acid in Sulfuric Acid–Dimethylamine New Particle Formation

Sandra K.W. Fomete[1,2], Jakub Kubečka[3], Jonas Elm[3], and Coty N. Jen[1,2*]

[1]Department of Chemical Engineering, Carnegie Mellon University, Pittsburgh, USA, 15213
[2]Center for Atmospheric Particle Studies, Carnegie Mellon University, Pittsburgh, USA, 15213
[3]Department of Chemistry, Aarhus University, Langelandsgade 140, 8000 Aarhus C, Denmark

*Corresponding author.
Email addresses: cotyj@andrew.cmu.edu (Coty N. Jen)

Section 1: Flow reactor baseline conditions with the PCC

To ensure clean and repeatable conditions in the flow reactor, daily baselines were obtained using an atmospheric pressure chemical ionization long time-of-flight mass spectrometer known as the Pittsburgh Cluster CIMS and a versatile water condensation particle counter (vWCPC). The baseline measurements are obtained by varying the flow rate of nitrogen over the malonic acid and sulfuric acid reservoirs in order to vary the malonic and sulfuric acid concentrations in the reactor. During these baselines, the total reactor flow rate is kept at 4.5 sLpm. The relative humidity (RH) in the reactor was kept constant at ~20 % in a temperature-controlled room at 300–303 K. The PCC, which was connected in-line to the flow reactor, measured the concentrations of sulfuric acid dimer ([SA_2]) and malonic acid dimers ([MaA_2]) for given sulfuric acid ([SA_1]) and malonic acid ([MaA_1]) monomer concentrations respectively injected into the reactor.

Figure S1(a) shows the malonic acid baselines with the PCC taken for 5 days over a period of 6 weeks. These baseline measurements relate the measured [MaA_2] detected at 207 m/z, 267 m/z, and 327 m/z, to [MaA_1] detected at 103 m/z by the PCC using acetate as the chemical ionization reagent ion. The malonic acid baselines show that [MaA_1] varied slightly across days. This is because the malonic acid reservoir had to be refilled often as the solution readily vaporized over the course of 3–4 days. Refilling the malonic acid reservoir during these few days led to slight differences in [MaA_1] for the same nitrogen flow rates over the reservoir. Nonetheless, the slope of [MaA_2] vs. [MaA_1] remained constant. A constant slope between the sulfuric acid dimer vs. acid monomer has previously been used to indicate the absence of stabilizing bases which generally enhances the acid dimer formation.[1–3]

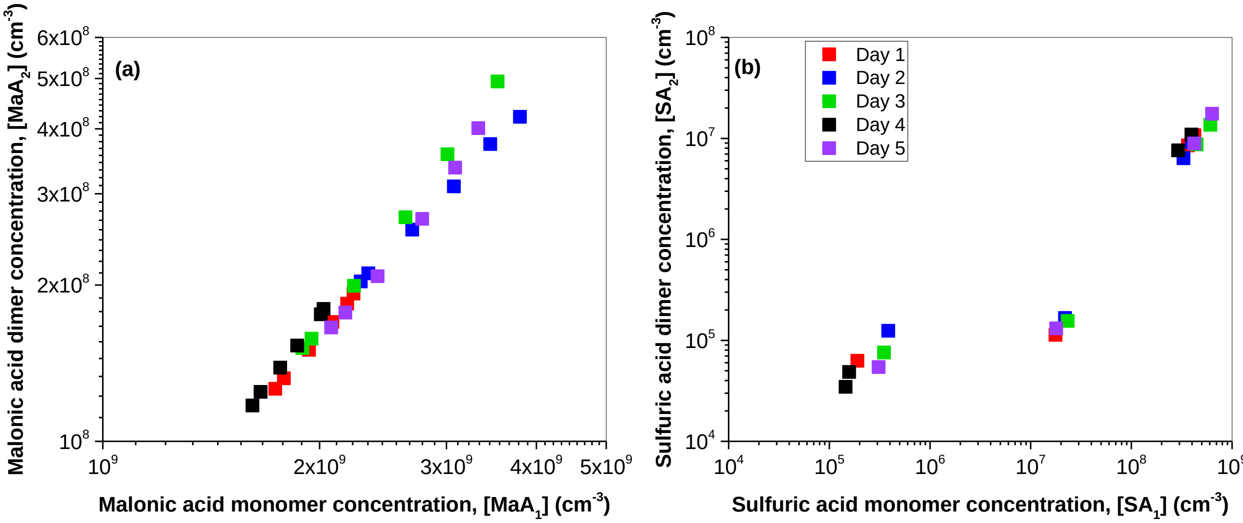

Figure S1: Baseline measurements showing: (a) malonic acid dimer concentration ([MaA_2]) vs. malonic acid monomer concentration ([MaA_1]), and (b) sulfuric acid dimer concentration ([SA_2]) versus sulfuric acid monomer concentration ([SA_1]) taken when [DMA] = 0 pptv on 5 different days during a 6-weeks period.

Figure S1(b) shows the sulfuric acid dimer concentration ($[SA_2]$) versus sulfuric acid monomer concentration ($[SA_1]$) taken on the same days as the malonic acid baselines shown in figure S1(a). The sulfuric acid baseline obtained by changing the injection flowrate of sulfuric acid between 10-100 sccm relies on consistent sulfuric acid monomer (97 m/z for acetate chemical ionization) and dimer (195 m/z) concentrations with no base added to the flow reactor. The total reactor flow rate did not change significantly during the sulfuric acid baseline since this small sulfuric acid flow was injected into the 4.5 sLpm flow of the reactor. Generally, the sulfuric acid baselines are consistent across the different days but deviate slightly at the lower sulfuric acid concentration. This is because it is difficult to obtain repeatable sulfuric acid concentrations in the reactor at the lower injection flow rates since the flow rates at the lower range of the mass flow controller are not very repeatable. The presence of base contaminants in the reactor would have led to deviations in measured $[N_2]$ was not observed in figure S1(b).

Section 2: Flow reactor baseline conditions with the vWCPC

Daily baseline measurements were also taken with the versatile water condensation particle counter (vWCPC, TSI 3789),[4] which has a 50% detection efficiency for 1 nm ions (geometric diameter). The vWCPC sampled from one of the side ports of the reactor as shown in Figure 1. The reactor and the vWCPC were operated such that the total flowrate in the reactor was kept constant for all the baseline measurements taken over 3 days as shown in Figure S2. The vWCPC baselines rely on monitoring the concentration of 1nm particles that were present in the reactor in the absence of added stabilizing bases like DMA. For a given sulfuric acid concentration, the malonic acid flowrate injected into the reactor was varied to observe the dependence of background particle concentrations on both sulfuric acid and malonic acid. As shown in Figure S2, the concentration of 1 nm particles generally does not vary with malonic acid flowrate injected into the reactor. However, at higher sulfuric acid concentrations, $[SA_1] = 8 \times 10^7$ cm^{-3} and $[SA_1] = 3 \times 10^8$ cm^{-3}, there is a small increase in the concentration of 1 nm particles at higher malonic acid concentrations. It is possible that at higher sulfuric acid injection flow rates, small amounts of base contaminants in the reactor could react with sulfuric acid to form 1 nm particle. Increasing the concentration of sulfuric acid injected into the reactor led to an increase in measured 1 nm particle concentrations. It is likely that sulfuric acid nucleated with residue DMA or other contaminants in the wick of the vWCPC to form these particles since background [DMA] in the reactor was less than 1 pptv.

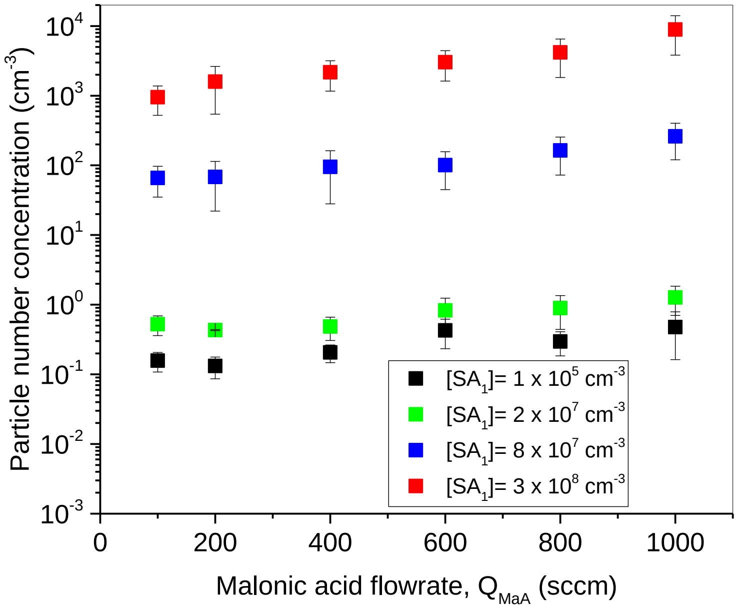

Figure S2: Baseline 1 nm particle number concentrations with [DMA] = 0 pptv measured with the versatile water condensation particle counter (vWCPC) over 3 days.

Section 3: Calculating sulfuric acid, malonic acid, dimethylamine concentrations from PCC measurements

The concentrations of malonic acid, sulfuric acid, and nucleated sulfuric acid-DMA clusters in the sample flow were measured in negative ion mode with the PCC using acetate as the chemical ionization reagent ion. The resulting reagent ions for acetate are $H_2O \cdot CH_3CO_2^-$(~ 6 x 10^3 Hz), $CH_3CO_2H \cdot CH_3CO_2^-$(~ 1 x 10^5 Hz), and $CH_3CO_2^-$ (~ 6 x 10^4 Hz) The DMA concentrations in the sample flow are measured in positive ion mode using hydronium ions, $(H_2O)_{1-2} \cdot H_3O^+$. In order to obtain concentrations of these nucleation precursors from the measured PCC signals, rate expression of the corresponding ions can be written as previously shown by Fomete et al. (2022).[5] The concentration of the sulfuric acid monomer, ($[SA_1]$) can be calculated from the signal ratio of HSO_4^- to the sum of the acetate reagent ion signals as shown below:

$$\frac{S_{HSO_4^-}}{S_{reagent}} = z_i k_1 [SA_1] t_{CI} \qquad (1)$$

Where, z_i is the factor describing the mass transmission efficiency discrimination of the measured HSO_4^- relative to the acetate reagent ion and t_{CI} is the chemical ionization reaction time. In this study, the mass-dependent transmission efficiencies were obtained from Henritizi et al.[6] for a time-of-flight chemical ionization mass spectrometer with a similar mass filter as the PCC. k_1, the forward rate constant for reaction of acetate with sulfuric acid was previously measured to be 4.6 x 10^{-9} cm^3 s^{-1}.[5] $S_{reagent}$ for acetate is the sum of the signals at 59 m/z ($CH_3CO_2^-$), 77 m/z ($H_2O \bullet CH_3CO_2^-$), and 119 m/z ($CH_3CO_2H \bullet CH_3CO_2^-$).

Equation 1 can also be used to solve for the malonic acid monomer concentration ($[MaA_1]$) using the ratio of the corresponding malonate ion signal ($C_3H_2O_4^-$) to the sum of the acetate reagent ion signals. Suppose malonic acid is ionized by acetate at the same rate constant as sulfuric acid assumed in this study (k_1 = 4.6 x 10^{-9} cm^3 s^{-1}). In that case, the malonic acid concentrations

calculated here represent the upper limit. The concentration of DMA can also be calculated using the signal ratio of the protonated DMA ion ($(CH_3)_2NH_2^+$) to the sum of the signals of the hydronium ions ($H_3O^+, H_2O \cdot H_3O^+,$ and $(H_2O)_2 \cdot H_3O^+$).

The chemical ionization reaction time in Equation 1, t_{CI} is given by:

$$t_{CI} = \frac{Distance\ between\ source\ plate\ and\ PCC\ inlet\ orifice\ (cm)}{electric\ field\ strength\ accross\ inlet\ \left(\frac{V}{cm}\right) * reagent\ ion\ mobility\ \left(\frac{cm^2}{s.V}\right)} \quad (2)$$

For the sulfuric acid malonic measurements, the t_{CI} was ~ 25 ms based on a 4.8 cm plate distance between the source and PCC inlet orifice, 84 V/cm electric field strength, and an acetate ion mobility of 2.32 cm^2/ s•V.[7]

Section 4: The cluster structures and thermodynamic properties

Table S1: The list of standard binding free energies at 298.15 K with all corrections applied (i.e., electronic energy correction, monomer symmetry correction, quasi-harmonic correction, and anharmonicity correction) for all computationally studied clusters.

Cluster	ΔG [kcal/mol]
1SA	0.00
1DMA	0.00
1MaA	0.00
2SA	-5.73
2DMA	5.48
1DMA1SA	-11.40
1DMA2SA	-29.28
2DMA1SA	-14.99
2DMA2SA	-45.00
2DMA3SA	-58.95
3DMA2SA	-50.01
1MaA1SA	-5.61

Cluster	ΔG [kcal/mol]
1MaA2SA	-12.36
2DMA1MaA	-3.28
1DMA1MaA1SA	-19.69
1MaA3SA	-18.13
3DMA1MaA	-1.71
2DMA1MaA1SA	-33.19
1DMA1MaA2SA	-34.13
2DMA1MaA2SA	-52.81
1DMA1MaA3SA	-45.35
3DMA1MaA1SA	-34.24
3DMA1MaA2SA	-65.45
2DMA1MaA3SA	-67.85

The cluster thermodynamic properties used for ACDC simulations are presented in Table S1. The structure of the MaA_1•SA_1 heterodimer cluster is shown in Figure S3. Other properties as well as other structures for the several lowest binding free energy configurations of each cluster can be downloaded from the GitHub repository (under Kubecka and Fomete folders):

https://github.com/elmjonas/ACDB/database_v2/DLPNO_vnw16/

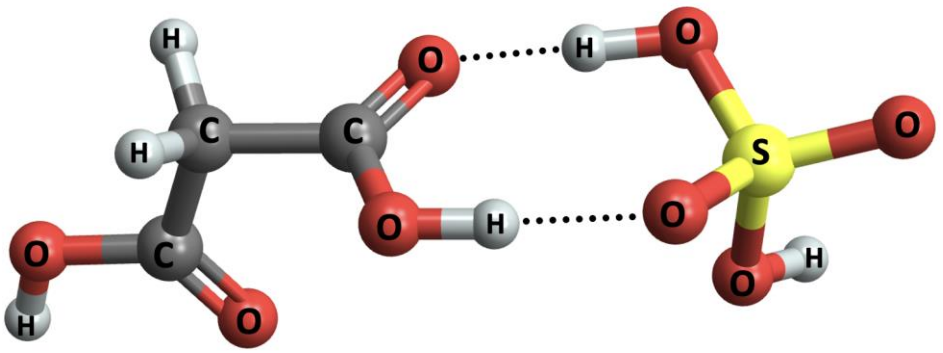

Figure S3: The structure of the $MaA_1 \bullet SA_1$ heterodimer showing the ability of hydrogen bonding between the two compounds.

Section 5: The results of ACDC simulations

Figure S4 show the concentrations of the MaA–SA dimer, the SA dimer, and all clusters containing two SA as a function of malonic acid concentration after the 10 s ACDC simulations. Figure S5 shows the particle formation rates at the end of the simulation. Clearly, the theoretical calculations and modelling do also suggest that MaA does not affect the NPF of SA-DMA system.

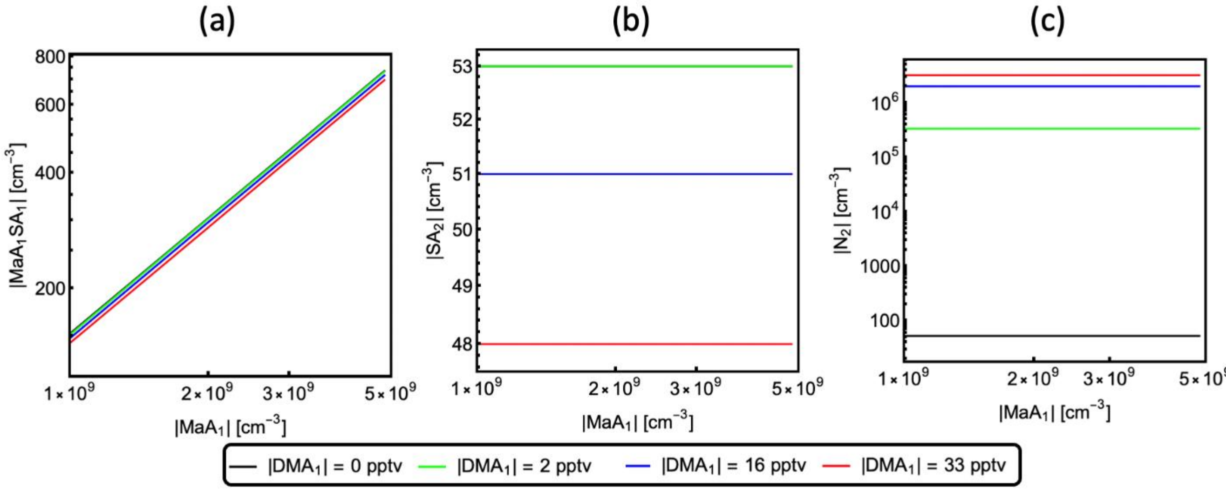

Figure S4: Concentrations of (a) MaA–SA dimer, (b) SA dimer, and (c) all clusters containing two SA as a function of malonic acid concentration after the 10 s ACDC simulations. Note that the green line almost overlaps the black line in the figures (a) and (b).

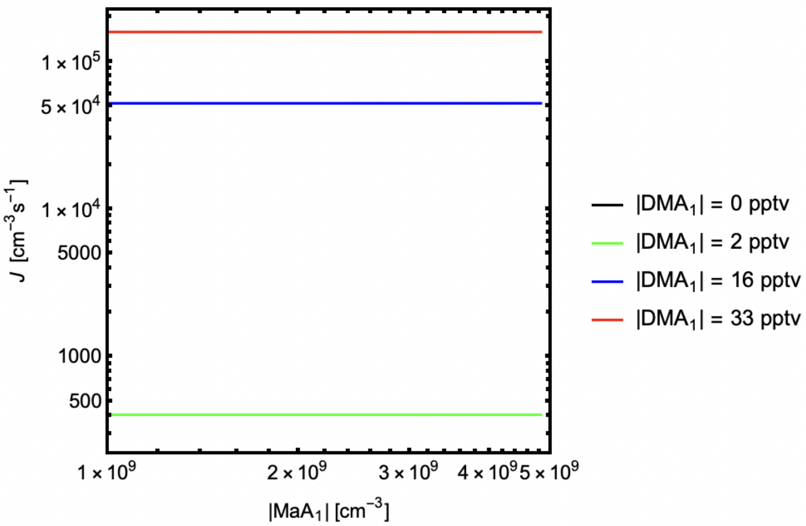

Figure S5: The new particle formation rate, *J*, as a function of different [MaA_1] and [DMA_1] after the 10 s ACDC simulations.

References

(1) Fomete, S. K. W.; Johnson, J. S.; Casalnuovo, D.; Jen, C. N. A Tutorial Guide on New Particle Formation Experiments Using a Laminar Flow Reactor. *Journal of Aerosol Science* **2021**, *157*, 105808. https://doi.org/10.1016/j.jaerosci.2021.105808.

(2) Jen, C. N.; McMurry, P. H.; Hanson, D. R. Stabilization of Sulfuric Acid Dimers by Ammonia, Methylamine, Dimethylamine, and Trimethylamine. *Journal of Geophysical Research: Atmospheres* **2014**, *119* (12), 7502–7514. https://doi.org/10.1002/2014JD021592.

(3) Jen, C. N.; Zhao, J.; McMurry, P. H.; Hanson, D. R. Chemical Ionization of Clusters Formed from Sulfuric Acid and Dimethylamine or Diamines. *Atmos. Chem. Phys.* **2016**, *16* (19), 12513–12529. https://doi.org/10.5194/acp-16-12513-2016.

(4) Hering, S. V.; Lewis, G. S.; Spielman, S. R.; Eiguren-Fernandez, A.; Kreisberg, N. M.; Kuang, C.; Attoui, M. Detection near 1-Nm with a Laminar-Flow, Water-Based Condensation Particle Counter. *Aerosol Science and Technology* **2017**, *51* (3), 354–362. https://doi.org/10.1080/02786826.2016.1262531.

(5) Fomete, S. K. W.; Johnson, J. S.; Myllys, N.; Neefjes, I.; Reischl, B.; Jen, C. N. Ion–Molecule Rate Constants for Reactions of Sulfuric Acid with Acetate and Nitrate Ions. *J. Phys. Chem. A* **2022**. https://doi.org/10.1021/acs.jpca.2c02072.

(6) Heinritzi, M.; Simon, M.; Steiner, G.; Wagner, A. C.; Kürten, A.; Hansel, A.; Curtius, J. Characterization of the Mass-Dependent Transmission Efficiency of a CIMS. *Atmos. Meas. Tech.* **2016**, *9* (4), 1449–1460. https://doi.org/10.5194/amt-9-1449-2016.

(7) Dwivedi, P.; Matz, L. M.; Atkinson, D. A.; Herbert H. Hill, J. Electrospray Ionization-Ion Mobility Spectrometry: A Rapid Analytical Method for Aqueous Nitrate and Nitrite Analysis. *Analyst* **2004**, *129* (2), 139–144. https://doi.org/10.1039/B311098B.
